# The Metabolism in Osteogenesis Imperfecta with Special Reference to Calcium

**Published:** 1914-07

**Authors:** 


**The Metabolism in Osteogenesis Imperfecta with Special Reference to Calcium.** Bookman, in the American Journal of Diseases of Children, June, 1914, reports a case from the service of Koplik at the Mount Sinai Hospital, in an infant of 10 weeks in whom the metabolism experiment, the two periods of which extended over six days, each seemed to prove that the calcium retention was below the normal, and that variations in the course of the disease caused changes in the calcium balance, also that the administration of phosphorus in cod liver oil, and especially of calcium lactate acted favorably.—C. G. L-W.

